# Association of only-child status and household pet ownership with attention-deficit/hyperactivity disorder among Chinese preschool children: a population-based study

**DOI:** 10.3389/fpubh.2024.1450216

**Published:** 2025-02-11

**Authors:** Yuying Zhang, Shuangyan Qiu, Vivian Yawei Guo, Weiqing Chen, Xiaomei Han, Weikang Yang

**Affiliations:** ^1^Department of Child Healthcare, Shenzhen Longhua Maternity and Child Healthcare Hospital, Shenzhen, China; ^2^School of Public Health, Sun Yat-sen University, Guangzhou, China

**Keywords:** attention-deficit/hyperactivity disorder (ADHD), preschool children, only-child status, pet ownership, joint effect

## Abstract

**Background:**

The associations of only-child status and household pet ownership with the risk of attention-deficit/hyperactivity disorder (ADHD) are inconclusive, and the joint effects of only-child status and household pet ownership on ADHD have not been thoroughly investigated.

**Methods:**

A population-based study was conducted in 2021 involving preschool children aged 3–6 years attending kindergartens in Longhua District, Shenzhen, China. Parents were invited to complete questionnaires providing information on socio-demographic and family-environmental factors. ADHD symptoms were assessed using the 26-item Swanson, Nolan, and Pelham Rating Scale as reported by parents.

**Results:**

This study included 63,282 children (mean age: 4.86 ± 0.85 years, 53.6% boys), representing 72.6% of all preschool children in this district in 2021. Among them, 34.4% were only-child and 9.6% were identified as having ADHD. Only-child status was associated with an increased risk of ADHD [adjusted odds ratio: 1.30 (95%CI: 1.23–1.38). Compared to children without a pet (cats or dogs) at ages 0–3 years, pet ownership at ages only 0–1 year, only 1–3 years, and both ages were associated with increased odds of ADHD: 1.59 (1.30–1.95), 1.58 (1.28–1.93), and 1.66 (1.42–1.92), respectively, after controlling for potential confounders. A significant interaction between pet ownership at only 1–3 years and only-child status was observed (adjusted P for interaction = 0.028). Similar findings were observed when the analyses were performed separately for boys and girls.

**Conclusion:**

Both only-child status and household pet ownership are associated with an increased risk of ADHD; however, the detrimental effect of pet ownership appears to be mitigated among only children when pet exposure occurs at ages 1–3 years, providing new insight into reducing family-related risk factors of ADHD.

## Introduction

Attention-deficit/hyperactivity disorder (ADHD) is the most prevalent neurodevelopmental disorder, affecting approximately 7.2% of children globally (1, 2). ADHD is characterized by hyperactivity, inattention, and impulsivity at developmentally inappropriate levels (3), leading to impaired education achievement, poor peer relationships, and an increased risk of other mental health disorders (4–6). Although ADHD is largely genetically inherited (70%), environmental factors also play a significant role in its etiology (7). Consequently, there is an urgent need to identify potential modifiable environmental risk factors for targeted prevention.

Siblings have been suggested to play a crucial role in children’s neurodevelopment, including the occurrence of ADHD (8–13). However, the impact of only-child status on ADHD risk remains inconclusive, and studies in this area are limited. Only children may experience distinct developmental environments compared to those with siblings (e.g., the absence of sibling interaction and increased attention from family members), which could potentially influence ADHD risk. A previous study involving 161 pairs of ADHD cases and non-ADHD controls revealed that being an only child was independently associated with an increased risk of ADHD diagnosis among Chinese children (11). Conversely, null findings were reported in the German Health Interview and Examination Survey for Children and Adolescents (KiGGS) study (*N* = 13,488) (12). In China, the one-child policy was in effect for approximately 40 years; although it was abolished in 2016, many families still have only one child. Given the significant burden of ADHD on individuals and families, further research is needed to elucidate the relationship between only-child status and ADHD, especially in light of policy changes in China.

In addition to only-child status, factors such as parental age, educational attainment, household income, and pet ownership collectively shape children’s upbringing environments. Among these, pet ownership has become increasingly common and may influence children’s neurodevelopment (13–16). The emotional benefits of pet attachment, such as providing emotional comfort, reducing stress, and fostering compassion and social skills, may contribute to neurodevelopmental advantages (14–20), particularly for only children lacking companionship. However, pets can also introduce environmental toxins (e.g., pesticides) into the home (21), which might increase the risk of ADHD (22). Currently, studies investigating the impact of early-life pet exposure on ADHD are limited, and existing findings remain inconsistent, with both positive and negative associations reported (17, 23). Therefore, it is essential to explore the impact of pet ownership on the risk of ADHD in children and whether pet companionship can mitigate the impact of being an only child.

Therefore, this population-based study investigated the impact of only-child status, pet ownership, and their interaction on the risk of ADHD in Chinese preschool children. Our findings aim to enhance the understanding of ADHD and help identify vulnerable children for targeted prevention.

## Methods

### Study design and participants

**Figure 1:**
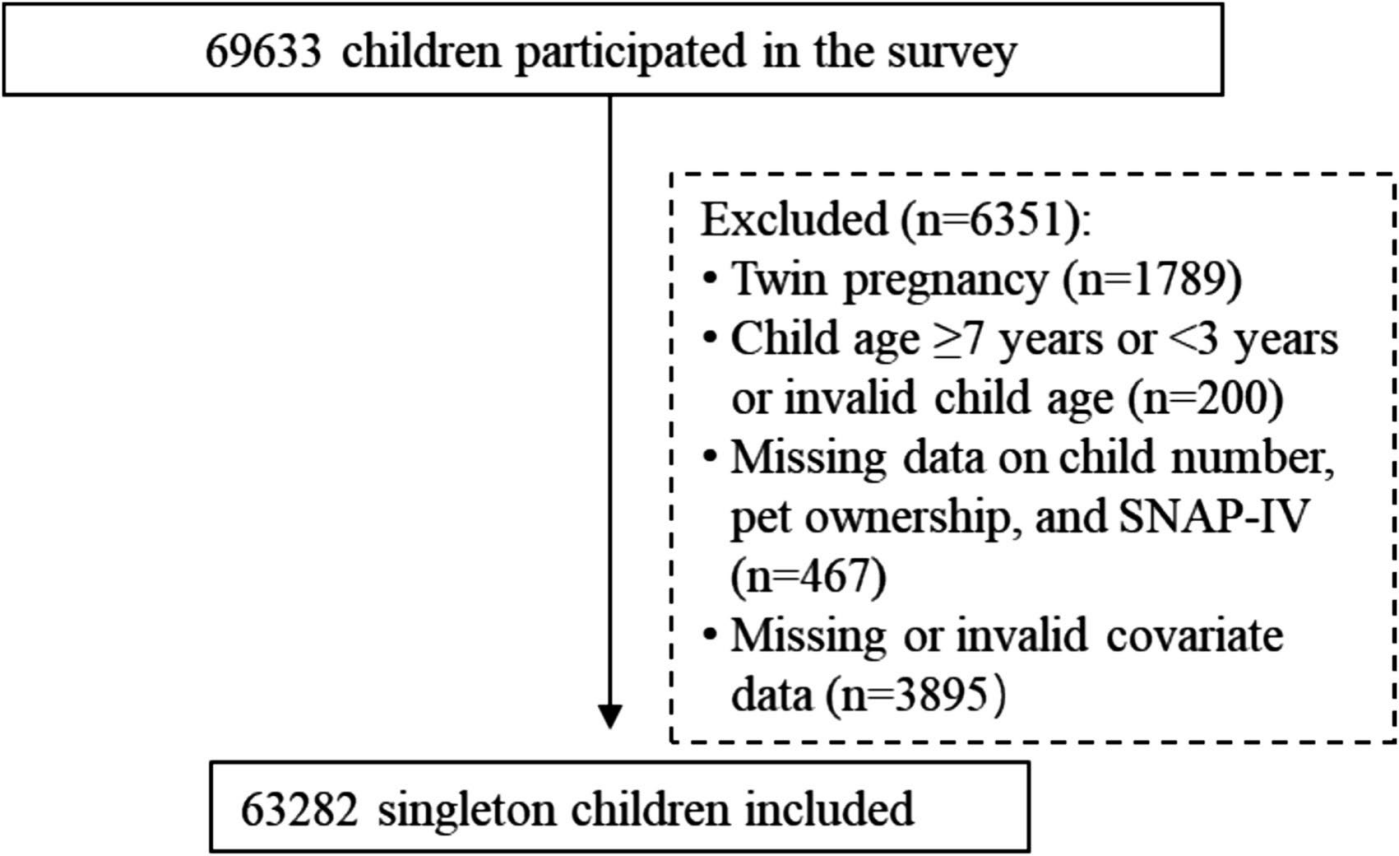
Participant selection.

This study utilized data from the 2021 wave of the Longhua Child Cohort Study, an annual population-based survey that evaluates the impact of family environment on children’s neurodevelopment (24, 25). In this survey, parents of participating children were invited to complete a structured questionnaire covering the socio-demographic information, children’s prenatal and early-life exposures (ages 0–3 years), and their neurodevelopment. The Chinese version of the questionnaire was administered via a mobile app specifically developed for this survey. A total of 87,081 children aged 3–6 years from 234 kindergartens were approached, and 69,633 participants completed the questionnaires which were filled out by their parents, representing 80.0% of preschool children in Longhua District. After excluding twin-born children and participants with missing or invalid data on child number, pet ownership, or other study variables, the final analysis included 63,282 children (Figure 1). The study was approved by the Ethics Committee of the Shenzhen Longhua Maternity and Child Health Care Hospital, and informed consent was obtained from all participants.

### Only-child status and pet ownership

Parents were asked to report the number of children in their family. Pet ownership was assessed through two self-reported questions: (1) “Did you own a pet cat or dog when this child was aged 0–1 years?” (2) “Did you own a pet cat or dog when this child was aged 1–3 years?” Responses to both questions were recorded as “yes” or “no.” Based on the answers, children were categorized into four groups: never owned a pet during ages 0–3 years, owned a pet only during ages 0–1 year, owned a pet only during age 1–3 years, and owned a pet during both age ranges.

### Measurement of ADHD symptoms

ADHD symptoms were measured using the 26-item Swanson, Nolan, and Pelham (SNAP) Revision 4 (SNAP-IV) scale, which has been validated in the Chinese population and is widely used for assessing ADHD symptoms (26). The SNAP-IV scale includes two subsets of ADHD symptoms (inattention and hyperactivity/impulsivity) based on DSM-IV criteria, as well as a subset for opposition/defiance. Each item is scored from ‘0’ (not at all) to ‘3’ (very often). Subset scores were calculated by summing the item scores, and children were classified as having borderline problems in inattention or hyperactivity/impulsivity if their respective subset score exceeded 13. The SNAP-IV scale demonstrated excellent reliability with a Cronbach’s *α* coefficient of 0.92 in this study.

### Covariates

Data on child age, sex, gestational age, mode of delivery, parental age and education background, household income, and marital status of biological parents were collected through a self-reported questionnaire completed by the children’s parents. Educational background was categorized into four levels: (1) primary or middle school, (2) high school, (3) college, and (4) graduate. Household income was divided into four groups: (1) <10,000 RMB/month, (2) 10,000–20,000 RMB/month, (3) 20,001–30,000 RMB/month, and (4) >30,000 RMB/month. Marital status was classified as married and unmarried/divorced groups. The mode of delivery was classified as vaginal or cesarean.

The 5-item Family Adaptation, Partnership, Growth, Affection, Resolve (F-APGAR) questionnaire was used to assess participants’ satisfaction with family functioning based on parameters of adaptability, partnership, growth, affection, and resolve. Responses were recorded on a 3-point scale (0 = hardly ever, 1 = sometimes, 2 = almost always), with a higher score indicating greater family support and better functioning (27).

### Statistical analysis

Data were presented as mean ± standard deviation (SD), number (%), or median (interquartile range) as appropriate. T-test, Mann-Whitney U-test, or chi-square test were used to compare characteristics between children with and without ADHD symptoms. Logistic regression analyses were performed to assess the associations between only-child status, pet ownership, and ADHD risk. Initially, associations were evaluated in a crude model, followed by a multivariate model adjusted for social-demographics and family-environmental confounders, including child age and sex, parental age, marital status, education, income, and family functioning. To further explore the interactive effects of pet ownership and only-child status on ADHD risk, children were divided into eight groups based on these two variables, and logistic regression was performed with group 1 (non-only children without pet ownership) serving as the reference group. Considering that ADHD is a sex-biased disorder, logistic regression analyses were further performed separately for boys and girls to evaluate potential sex-specific associations.

All analyses were performed using R software (version 4.2), with statistical significance set at a *p*-value of <0.05 (two-sided).

## Results

The characteristics of study participants are shown in Table 1. The mean age of the children was 4.86 ± 0.85 years, with 53.6% being boys. Among these children, 60,316 (95.3%) reported no pet ownership during ages 0–1 or 1–3 years. In contrast, 744 (1.2%) reported pet ownership only at ages 0–1 year, 786 (1.2%) only during ages 1–3 years, and 1,436 (2.3%) during both age ranges. Overall, 21,751 (34.4%) of the children were only children, and 6,049 (9.6%) were identified as having ADHD according to the SNAP-IV scale. Compared to those without ADHD, children with ADHD were younger, more likely to be boys, and had parents who were younger, less educated, and more likely to be unmarried or divorced. In addition, these families reported lower household income and family functioning. The gestational age and mode of delivery of these children did not differ significantly between groups. Pet ownership and only children were more prevalent among children in the ADHD group.

**Table 1:** Characteristics of participants.

	Overall	ADHD		*p*-value
		No	Yes	
Number	63,282	57,233 (90.4)	6,049 (9.6)	
Child age (years)	4.86 ± 0.85	4.86 ± 0.85	4.82 ± 0.86	0.002
Child sex				<0.001
Girls	29,387 (46.4)	27,269 (47.6)	2,118 (35.0)	
Boys	33,895 (53.6)	29,964 (52.4)	3,931 (65.0)	
Maternal age (years)	33.9 ± 4.4	34.0 ± 4.4	33.1 ± 4.4	<0.001
Paternal age (years)	36.1 ± 5.0	36.2 ± 5.0	35.2 ± 5.0	<0.001
Maternal education				<0.001
Primary or middle school	9,202 (14.5)	8,158 (14.3)	1,044 (17.3)	
High school	12,794 (20.2)	11,556 (20.2)	1,238 (20.5)	
College	39,192 (61.9)	35,580 (62.2)	3,612 (59.7)	
Graduate	2,089 (3.3)	1,937 (3.4)	152 (2.5)	
Paternal education				<0.001
Primary or middle school	8,213 (13.0)	7,276 (12.7)	937 (15.5)	
High school	12,958 (20.5)	11,656 (20.4)	1,302 (21.6)	
College	38,738 (61.3)	35,177 (61.6)	3,561 (58.9)	
Graduate	3,255 (5.2)	3,014 (5.3)	241 (4.0)	
Household income				<0.001
<10,000RMB/month	9,347 (14.8)	8,283 (14.5)	1,064 (17.6)	
10,000–20,000 RMB/month	21,915 (34.6)	19,704 (34.4)	2,211 (36.6)	
20,001–30,000 RMB/month	13,809 (21.8)	12,550 (21.9)	1,259 (20.8)	
>30,000 RMB/month	18,211 (28.8)	16,696 (29.2)	1,515 (25.0)	
Marital status of parents				<0.001
Married	62,666 (99.0)	56,694 (99.1)	5,972 (98.7)	
Unmarried or divorced	616 (1.0)	539 (0.9)	77 (1.3)	
Gestational age (weeks)	39.0 ± 1.6	39.0 ± 1.6	39.0 ± 1.7	0.45
Mode of delivery				
Vaginal	42,286 (66.8)	38,186 (66.7)	4,100 (67.8)	0.099
Cesarean	20,996 (33.2)	19,047 (33.3)	1,949 (32.2)	
F-APGAR score	7.0 [5.0, 10.0]	8.0 [5.0, 10.0]	6.0 [5.0, 8.0]	<0.001
Pet ownership				<0.001
No	60,316 (95.3)	54,726 (95.6)	5,590 (92.4)	
Only during age 0–1 year	744 (1.2)	627 (1.1)	117 (1.9)	
Only during ages 1–3 years	786 (1.2)	669 (1.2)	117 (1.9)	
During both age ranges	1,436 (2.3)	1,211 (2.1)	225 (3.7)	
Number of children (%)				<0.001
1	21,751 (34.4)	19,227 (33.6)	2,524 (41.7)	
2	36,771 (58.1)	33,597 (58.7)	3,174 (52.5)	
3	4,760 (7.5)	4,409 (7.7)	351 (5.8)	

ADHD, attention-deficit/hyperactivity disorder.

F-APGAR, The 5-item Family Adaptation, Partnership, Growth, Affection, Resolve (F-APGAR) scale. A higher score indicates a better family functioning.

As shown in Table 2, being an only child was significantly associated with increased odds of ADHD [odds ratio (OR) = 1.42 (95%CI: 1.34–1.49), *p* < 0.001]. This association remained significant [adjusted OR = 1.30 (95% CI: 1.23–1.38), *p* < 0.001] after adjustment for confounders including child age, child sex, maternal age, paternal age, maternal education, paternal education, marital status of parents, household income, and family functioning. Notably, an increasing number of children in a family was significantly associated with a decreased risk of ADHD. Compared to families with three children or above, the odds of ADHD for families with two children and only one child were 1.19 (1.06–1.33) and 1.65 (1.47–1.86), respectively, in the crude model; these odds remained significant in the adjusted model. Similar findings were obtained when the analyses were performed separately for boys and girls.

**Table 2:** Univariate and multivariate logistic regressions on the associations of only-child status and pet ownership with risk of ADHD.

	Crude model		Adjusted model*	
	Odds ratio	*p*-value	Odds ratio	*p*-value
Overall				
Only child (yes vs. no)	1.42 (1.34–1.49)	<0.001	1.30 (1.23–1.38)	<0.001
Number of children				
≥3	1.00 (reference)		1.00 (reference)	
2	1.19 (1.06–1.33)	0.003	1.22 (1.09–1.38)	0.001
1	1.65 (1.47–1.86)	<0.001	1.56 (1.39–1.77)	<0.001
Pet ownership				
No	1.00 (reference)		1.00 (reference)	
Only during age 0–1 year	1.83 (1.49–2.22)	<0.001	1.59 (1.30–1.95)	<0.001
Only during ages 1–3 years	1.71 (1.40–2.08)	<0.001	1.58 (1.28–1.93)	<0.001
During both age ranges	1.82 (1.57–2.10)	<0.001	1.66 (1.42–1.92)	<0.001
Boys				
Only child (yes vs. no)	1.44 (1.34, 1.54)	<0.001	1.33 (1.24, 1.43)	<0.001
Number of children				
≥3	1.00 (reference)		1.00 (reference)	
2	1.24 (1.07–1.44)	0.005	1.25 (1.08–1.46)	0.004
1	1.74 (1.50–2.02)	<0.001	1.63 (1.40–1.91)	<0.001
Pet ownership				
No	1.00 (reference)		1.00 (reference)	
Only during age 0–1 year	1.89 (1.46, 2.42)	<0.001	1.65 (1.26, 2.12)	<0.001
Only during ages 1–3 years	1.75 (1.34, 2.25)	<0.001	1.59 (1.21, 2.05)	<0.001
During both age ranges	1.91 (1.58, 2.29)	<0.001	1.71 (1.41, 2.06)	<0.001
Girls				
Only child (yes vs. no)	1.35 (1.23, 1.47)	<0.001	1.26 (1.15, 1.39)	<0.001
Number of children				
≥3	1.00 (reference)		1.00 (reference)	
2	1.10 (0.92–1.33)	0.287	1.19 (0.99–1.44)	0.066
1	1.47 (1.22–1.78)	<0.001	1.48 (1.22–1.81)	<0.001
Pet ownership				
No	1.00 (reference)		1.00 (reference)	
Only during age 0–1 year	1.78 (1.27, 2.44)	<0.001	1.51 (1.07, 2.08)	0.014
Only during ages 1–3 years	1.76 (1.28, 2.38)	<0.001	1.57 (1.13, 2.12)	0.005
During both age ranges	1.75 (1.37, 2.20)	<0.001	1.57 (1.22, 1.99)	<0.001

*Adjusted for child age, maternal age, paternal age, maternal education, paternal education, marital status, household income, family functioning score, and child sex were additionally adjusted in the overall model.

ADHD, attention-deficit/hyperactivity disorder.

Compared to those without pets at either age 0–1 or age 1–3 years, children with pets only during age 0–1 year, only during age 1–3 years, and during both age ranges were associated with increased odds of 1.83 (1.49–2.22), 1.71 (1.40–2.08), and 1.82 (1.57–2.10) for ADHD, respectively. The odds remained significant in the multivariate model. Similar findings were observed when analyses were performed separately for boys and girls.

We subsequently examined the interaction between only-child status and pet ownership. Compared to non-only child without pet ownership, pet ownership during either age 0–1 or age 1–3 years was significantly associated with an increased risk of ADHD, regardless of only-child status. Furthermore, only children were associated with increased odds of ADHD irrespective of pet ownership. A significant interaction was noted among only children with pet ownership only during ages 1–3 years (P for interaction = 0.028). When analyses were performed separately for boys and girls, similar findings were observed with significant interaction observed among girls but not boys (Table 3).

**Table 3:** Multivariate logistic regression evaluates the interaction of only-child status and pet ownership at different ages on the risk of ADHD.

	Only child		P for interaction
	No	Yes	
Overall			
Pet ownership			
No	1.00 (reference)	1.32 (1.24–1.40)^***^	
Only during age 0–1 year	1.59 (1.18–2.11)^**^	2.00 (1.50–2.64)^***^	0.827
Only during ages 1–3 years	1.91 (1.48–2.45)^***^	1.52 (1.07–2.12)^*^	**0.020**
During both age ranges	1.86 (1.52–2.27)^***^	1.83 (1.46–2.27)^***^	0.055
Boys			
Pet ownership			
No	1.0 (reference)	1.35 (1.25–1.45)^***^	
Only during age 0–1 year	1.72 (1.16–2.46)^**^	2.03 (1.40–2.87)^***^	0.622
Only during ages 1–3 years	1.81 (1.28–2.51)^**^	1.76 (1.12–2.65)^*^	0.239
During both age ranges	1.97 (1.52–2.52)^***^	1.89 (1.41–2.50)^***^	0.080
Girls			
Pet ownership			
No	1.00 (reference)	1.28 (1.16–1.41)^***^	
Only during age 0–1 year	1.40 (0.84–2.18)	2.01 (1.23–3.11)^**^	0.726
Only during ages 1–3 years	2.06 (1.39–2.96)^***^	1.21 (0.64–2.08)	**0.028**
During both age ranges	1.70 (1.20–2.35)^**^	1.76 (1.22–2.47)^**^	0.393

Adjusted for child age, maternal age, paternal age, maternal education, paternal education, marital status, household income, and family function score; in the overall model, child sex was additionally adjusted.

ADHD, attention-deficit/hyperactivity disorder. Bold values are significantly findings.

## Discussion

The current study found that both pet ownership and being an only child were associated with an increased risk of ADHD among Chinese preschool children. However, the detrimental impact of pet ownership appeared to be mitigated among only children when exposure to pets occurred during ages 1–3 years.

The association between being an only child and the risk of ADHD remains inconclusive. The KiGGS study, which included 13,488 children and adolescents, reported no significant associations between the number of siblings and ADHD risk (12). In contrast, a case–control study of 161 pairs of Chinese children with ADHD and matched controls unveiled a positive association between being an only child and ADHD diagnosis (11). Our study also identified an increased likelihood of ADHD among only children. The mechanisms underlying this correlation remain unclear. One possible explanation is that only children often receive more attention from family members, which may inadvertently interrupt their activities and affect their ability to concentrate. In addition, only children may lack home-based playmates, potentially hindering their neurodevelopment. Furthermore, heightened parental concern for only children may lead to increased reporting of behavioral issues during assessments. It is also worth noting that due to the cross-sectional nature of the current study, we cannot rule out the possibility of reverse causality. For instance, parents of children exhibiting early signs of ADHD may have chosen not to have additional children. Given these complexities, further investigations into the causal relationship between only-child status and ADHD are imperative.

While there is evidence suggesting that pet attachment can positively influence children’s neurodevelopment, the relationship between early-life pet exposure and ADHD risk remains ambiguous, with a limited number of studies presenting mixed findings. A prior study involving 4,860 children from 2 German birth cohorts found that pet ownership at any point from birth to age 10 years was linked to higher scores for emotional symptoms and hyperactivity/impulsivity by age 10 years (28). Similarly, the 2003 California Health Interview Survey identified a positive correlation between allowing dogs or cats into the house and ADHD risk (29). However, this association diminished and became statistically insignificant after doubly robust adjustments for confounding factors, using models that incorporated both propensity score variables and propensity score weights (29). A U.S. birth cohort study indicated that maternal prenatal dog ownership was positively linked to ADHD in boys but not girls (23). In contrast, another cohort study reported a reduced risk of mental health disorders, including ADHD, in adolescents who had childhood exposure to pet dogs or cats (17). In the present study, pet cat or dog ownership during early childhood was significantly associated with an increased risk of ADHD. Pets may influence children’s neurodevelopment in various ways, including potential alterations in the gut microbiome that could affect ADHD risk via the gut–brain axis (30–33). Moreover, pets might introduce environmental toxins (e.g., pesticides) into the home, posing additional ADHD risks (21, 22). Thus, further studies are warranted to elucidate the mechanisms underlying the association between early-life pet exposure and ADHD risk.

Interestingly, the negative effects of pet ownership appeared to be mitigated for only children when pet exposure occurred between the ages of 1 and 3 years. We hypothesize that pet cat or dog companionship may provide emotional comfort, compensating for the absence of siblings. This effect seems particularly evident during the critical developmental window of age 1 to 3 years, a period when children gradually transition from primarily interacting with parents to engaging with peers, acquiring fundamental social skills. Further research is essential to validate our findings and explore the underlying mechanisms.

### Limitations

The primary strength of this study is its population-based design, encompassing a substantial sample size of over 60,000 children. However, several limitations must be considered when interpreting the findings. First, the reliance on self-reported data induces potential recall and self-report biases. Second, due to the cross-sectional design, which is susceptible to reverse causality, causal inference is limited. Third, although birth order has been linked to ADHD risk, this study did not collect the birth order data, precluding further investigation into its fluence. In addition, we did not differentiate between pet cats and dogs, despite evidence suggesting they may have distinct impacts on children’s neurodevelopment. Furthermore, we did not collect data on ownership of other pets (e.g., birds and fish) which may also influence ADHD risk. Moreover, we lacked detailed information on the duration of children’s interactions with their pets, which could mediate the observed effects of pet exposure. Our dataset also lacks information on the family history of ADHD, and given the heritable nature of ADHD, it is conceivable that our findings regarding pet ownership and ADHD could be influenced by unaccounted family history or genetic predisposition. Despite these limitations, this study provides valuable insights into the relationship between pet ownership, only-child status, and ADHD risk. Future longitudinal studies are warranted to address these limitations and establish causal relationships.

## Conclusion

This population-based study identifies an association between only-child status, exposure to pet dogs/cats, and ADHD risk among children in China. Families and healthcare providers should be aware of these potential risks. It is essential for parents, particularly those of only children, to adopt appropriate parenting strategies, including providing proper supervision and offering children more companionship to support their emotional and social development. Further research utilizing prospective longitudinal birth cohorts is necessary to elucidate the causal relationships between family-environmental factors and ADHD risk. Such studies may provide a foundation for developing effective interventions aimed at mitigating family-related risk factors and preventing ADHD in children.

## Data Availability

The data supporting the findings of this study are available from the corresponding author upon reasonable request.
